# Beta-Lactam Antibiotic Discrimination Using a Macromolecular Sensor in Water at Neutral pH

**DOI:** 10.3390/s21196384

**Published:** 2021-09-24

**Authors:** Yifei Xu, Marco Bonizzoni

**Affiliations:** 1 Department of Chemistry and Biochemistry, The University of Alabama, Tuscaloosa, AL 35487, USA; xxu56@crimson.ua.edu; 2 Alabama Water Institute, The University of Alabama, Tuscaloosa, AL 35487, USA

**Keywords:** β-lactam antibiotics, pattern-based recognition, linear discriminant analysis, fluorescence, PAMAM dendrimer

## Abstract

Penicillins and cephalosporins belong to the β-lactam antibiotic family, which accounts for more than half of the world market for antibiotics. Misuse of antibiotics harms human health and the environment. Here, we describe an easy, fast, and sensitive optical method for the sensing and discrimination of two penicillin and five cephalosporin antibiotics in buffered water at pH 7.4, using fifth-generation poly (amidoamine) (PAMAM) dendrimers and calcein, a commercially available macromolecular polyelectrolyte and a fluorescent dye, respectively. In aqueous solution at pH 7.4, the dendrimer and dye self-assemble to form a sensor that interacts with carboxylate-containing antibiotics through electrostatic interaction, monitored through changes in the dye’s spectroscopic properties. This response was captured through absorbance, fluorescence emission, and fluorescence anisotropy. The resulting data set was processed through linear discriminant analysis (LDA), a common pattern-base recognition method, for the differentiation of cephalosporins and penicillins. By pre-hydrolysis of the β-lactam rings under basic conditions, we were able to increase the charge density of the analytes, allowing us to discriminate the seven analytes at a concentration of 5 mM, with a limit of discrimination of 1 mM.

## 1. Introduction

Antibiotics are used for the treatment of human and animal diseases. They are also added to animal feed to improve growth rates and feed efficiency [1,2,3]. However, the overuse of antibiotics has given rise to a number of multi-resistant bacteria, and the residues in food may cause serious allergy problems [4]. Penicillins and cephalosporins, members of the β-lactam antibiotic family, comprise about 65% of the world market for antibiotics [5]. Thus, there is a need for the detection of these antibiotics appearing in foods and environmental samples.

**Scheme 1:**
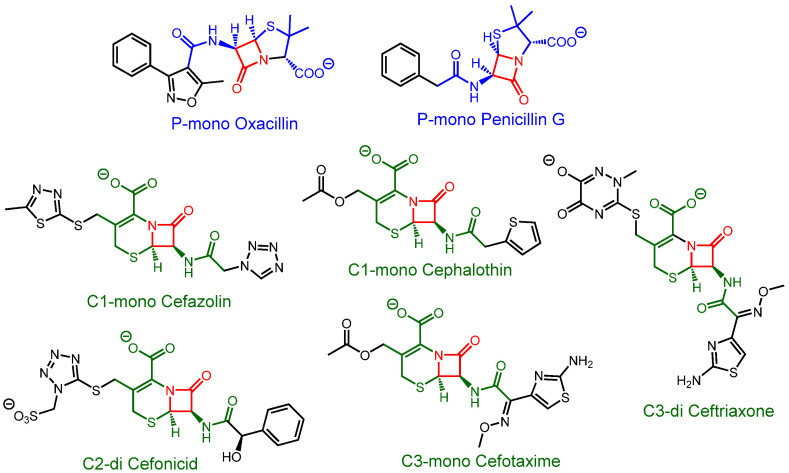
Structures of the penicillins and cephalosporins of interest (red: β-lactam ring, blue: penicillin core structure, green: cephalosporin core structure), shown in their most likely protonation state in water at pH 7.4. P: penicillin, C: cephalosporin generation number, mono: monoanion, di: dianion.

We will discuss work leading to the discrimination of five cephalosporins and two penicillins whose structures are shown in Scheme 1, where the structure in red is the β-lactam ring, while green and blue stands for the core structure of cephalosporins and penicillins, respectively. For easier reference to the analytes, here we use P to stand for penicillins and C for cephalosporins; numbers stand for the cephalosporin generation number; monoanions and dianions are marked as “mono” and “di”, respectively. We also introduced two controls in the study, an organic dicarboxylate (1,4-benzenedicarboxylate, terephthalate) and a monocarboxylate (benzoate). Several applications can benefit from a system that can qualitatively recognize the identity of common β-lactam antibiotics rapidly and easily, such as testing antibiotic residues in food (for example, in milk) to prevent food allergies; measuring the type and amount of antibiotics in sewer water, to get an overall picture of antibiotic consumption in an area; and comparing the downstream and upstream antibiotic content of a pasture to test for abusive administration of antibiotics to farm animals.

Counterfeiting of antibiotics is also a global health issue. Structures reported to be counterfeited include cefazolin, cefotaxime, and ceftriaxone (included as analytes in this study) [6]. Common problems with counterfeit antibiotics include reduced concentration of the active principle, reduced stability, altered composition, and introduction of impurities [7]. Common detection methods for β-lactam antibiotics include the European Union Four-Plate Test (EU4pt) [8,9], the *Bacillus stearothermophilus* Disc Assay (BsDA) [10,11], the Nouws Antibiotic Test (NAT) [12,13], and the PremiTest [14,15]. The currently available tests typically rely on the inhibition of bacterial growth. Unfortunately, such growth inhibition methods are generally non-selective (i.e., they do not identify the specific antibiotic being detected, but only its biologically effective concentration) and cumbersome to use [16]; they also display great differences in sensitivity across different classes of antibiotics [17]. The present work provides a selective method of identification and discrimination of antibiotics, even with similar chemical structure. Furthermore, the test described here is simpler to use and faster than the microbial growth inhibition ones in common use. Towards this goal, we report here on the use of a sensing complex (comprised of a host molecule and a fluorophore) requiring only simpler techniques and detection systems for the discrimination of β-lactam antibiotics, namely optical spectroscopy methods coupled with industry-standard microwell plates. In contrast to existing methods in common use, this approach directly detects distinctive chemical features of the antibiotics under study, rather than their biological effects (i.e., inhibition of bacterial growth, an indirect detection paradigm).

**Scheme 2:**
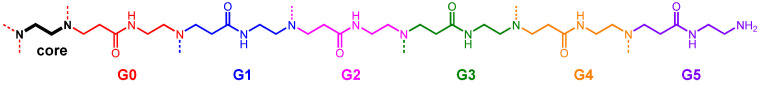
One branch of an amine-terminated fifth generation poly (amidoamine) (PAMAM G5) dendrimer with ethylene diamine core. Each generation ends with an amine group and branches out twice; at each branching point, one branch is shown in the scheme and the other identical branch is represented by the dashed line.

Our group has previously reported that amine-terminated poly(amidoamine) (PAMAM) dendrimers can be used as supramolecular hosts for anionic organic compounds in array sensing applications using simple conditions and instrumentation. The requirement for analytical selectivity of each component receptor is greatly diminished for these applications [18]. PAMAM dendrimers are commercially available, water-soluble globular hyperbranched polymers whose surface presents a high-density, regular array of primary ammonium groups that allow these macromolecules to bind smaller guests in solution through non-covalent interactions [19,20]. Such hosts carry positive charges in water near neutral pH, by virtue of the protonation of half of the amine groups [21], so they predominantly establish hydrogen bonding and electrostatic interactions with a variety of anionic species. We report here on the use of these supramolecular anion receptors in the analytical discrimination of the carboxylate-containing antibiotics shown above in water. In this work, fifth generation (G5) PAMAM dendrimers (shown in Scheme 2) were used, because they afford an excellent balance between high number and density of functional groups, and affordable cost. About half of the 128 surface amine groups of a G5 PAMAM are protonated and carry a positive charge at pH 7.40 in water, providing a binding site for the carboxylate antibiotics through electrostatic and hydrogen bonding interaction.

**Scheme 3:**
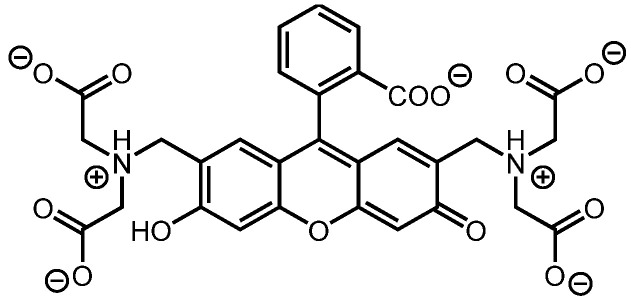
Structure of calcein in its most likely protonation state in water at pH 7.4.

However, because both the selected PAMAM receptors and the antibiotics are spectroscopically silent in the visible region of the spectrum, those interactions are hard to monitor. We circumvented this problem by building an indicator displacement assay (IDA) to transform these silent receptors into full-fledged chemical sensors, responsive to the presence of their analytical targets by changes in their macroscopic properties. In aqueous HEPES (2-[4-(2-hydroxyethyl)piperazin-1-yl] ethanesulfonic acid) buffer under neutral conditions, the complex between an anionic organic dye and a PAMAM G5 dendrimer was first formed. Then, with the introduction of a carboxylate analyte, the latter formed its complex with PAMAM and caused dye displacement; the binding between dendrimer and analyte could therefore be monitored through the resulting spectroscopic behavior of the dye. We previously reported a sensor system built from PAMAM G5 and a commercially available dye, calcein (see Scheme 3), that was successful for the discrimination and quantitation of biologically relevant carboxylates in aqueous solution at pH 7.4 [22]. The β-lactam antibiotics targeted here all contain one carboxylate group in their core structure which can be a point of interaction with the PAMAM-calcein sensor, so, in this study, we expand the scope of that system towards the sensing of carboxylate-containing antibiotics, using similar conditions to those reported previously ([calcein] = 6.36 μM and [PAMAM G5] = 2.13 μM in 50 mM aqueous HEPES buffer at pH 7.4). However, preliminary testing led us to implement a crucial improvement on our previously reported method, centered on sample pre-treatment. This allowed us to obtain effective discrimination of the antibiotic analytes, as discussed below.

Benchtop titrations were first carried out to determine whether the selected antibiotics would bind to PAMAM dendrimers, and to select the most appropriate working concentration for discrimination studies. Discrimination studies were then carried out using 384-well microplates and a plate reader, and linear discriminant analysis (LDA) was used for multivariate data processing.

## 2. Materials and Methods

A fifth-generation, amine-terminated poly(amidoamine) (PAMAM) dendrimer with a 1,2-diaminoethane core was manufactured by Dendritech, Inc. (Midland, MI, USA) and purchased as a solution in methanol with an exact concentration of 1.419 mM. The final solution used for all experiments was obtained by dilution of this stock with 50 mM HEPES buffer (see below) and contained a negligible amount of methanol (<0.8%). Dye stock solution was prepared from calcein disodium salt purchased from Sigma Aldrich (St. Louis, MO, USA). Analyte solutions were prepared from terephthalic acid, benzoic acid, and ceftriaxone sodium salt hemiheptahydrate purchased from Acros (Geel, Belgium); cefazolin sodium salt and cefonicid sodium salt purchased from CHEM-IMPEX (Wood Dale, IL, USA); penicillin G sodium salt purchased from Alfa Aesar (Haverhill, MA, USA); and cephalothin, oxacillin sodium salt monohydrate, and cefotaxime sodium salt purchased from TCI (Tokyo, Japan). A buffer of 50 mM 4-(2-hydroxyethyl) piperazine-1-ethanesulfonic acid (HEPES) was prepared from HEPES purchased from IBI Scientific (Dubuque, IA, USA). All prepared solutions were adjusted to pH 7.4 using solutions of NaOH, purchased from Fisher Scientific (Waltham, MA, USA), and HCl, purchased from BDH Aristar (Dubai, UAE). Nunc 384-well polystyrene black-wall plates with clear flat well bottoms were purchased from Thermo Scientific (Waltham, MA, USA). All materials were used as received.

Absorbance titrations were carried out on a HP 8452A diode array spectrophotometer, measuring from 230 nm to 800 nm with a 2 nm wavelength resolution. Fluorescence emission and fluorescence anisotropy titration were carried out on an ISS PC1 spectrofluorometer, with a broad-spectrum high-pressure xenon lamp (CERMAX, 300 W) as an excitation light source, manual calibrated slits, and excitation correction by a rhodamine B quantum counter with a dedicated detector. The emission light detector was a Hamamatsu red-sensitive PMT operating in photon-counting mode. High-aperture Glan–Thompson calcite polarizers were used for fluorescence anisotropy measurement. For all titration experiments, an external circulating water bath was used to maintain the cuvette temperature at 25 °C.

Microwell plate-based discrimination studies were carried out on a Biotek Synergy II multimode plate reader, with a tungsten lamp light source. A monochromator was used for absorbance measurements, different bandpass filters were used for fluorescence emission measurements, and plastic sheet polarizers were added for fluorescence anisotropy measurements. A “top-detected” mode was used for all fluorescence measurements, whereby a dichroic mirror was automatically positioned between the emission source and sample wells to block excitation light from reaching the detector.

All experiments were performed in 50 mM HEPES buffer at pH 7.4. pH was measured with a combined glass electrode and adjusted by appropriate addition of NaOH or HCl solutions. The concentrations for anion binding titrations were as follows: [calcein] = 6.36 µM with [G5] = 2.13 µM. For hydrolysis of antibiotics, analytes were dissolved in water at pH 10 or pH 12 to reach a 0.1 M concentration and shaken for two minutes at the elevated pH, then the solution’s pH was adjusted to pH 7.4 for spectroscopic measurements. The concentrations for qualitative discrimination experiments were as follows: [calcein] = 6.36 µM with [G5] = 2.13 µM, [analytes] = 5.0 mM. The concentrations for limit of discrimination experiments were as follows: [calcein] = 6.36 µM with [G5] = 2.13 µM, [analytes] = 1.0 mM.

Antibiotic binding experiments were carried out by adding a “titrant” solution containing one analyte and [calcein•PAMAM] complex (concentration listed above) into 2 mL of a “cuvette” solution containing the same concentration of [calcein•PAMAM] complex. Therefore, concentration of the calcein dye and PAMAM dendrimer remained constant during titration to ensure that any observed changes derived from a chemical process within the sample rather than from dilution artifacts. All titrations were performed in a 1 cm quartz cuvette.

For qualitative discrimination experiments, each analyte sample was prepared in 32 replicates. Solutions were prepared in 50 mM HEPES buffer, and 100 µL of each sample was deposited into the wells by hand using Eppendorf Research multichannel pipettors. Absorbance, fluorescence emission, and anisotropy were measured through a plate reader. Data processing was carried out by Linear Discriminant Analysis (LDA) algorithms, and data validation was based on a bootstrap approach using PCA analysis [23]. All data processing and analysis was carried out using Wolfram Mathematica version 12.0.

## 3. Results

### 3.1. Indicator Displacement Setup: Binding of Calcein Dye to PAMAM G5 Dendrimer

**Figure 1:**
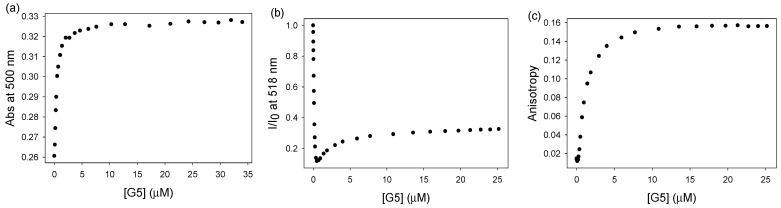
Titration of G5 into calcein to form [calcein•PAMAM] complex in solution. (**a**) Absorbance; (**b**) fluorescence emission intensity; (**c**) fluorescence anisotropy. [calcein] = 6.36 μM, [PAMAM G5] = 2.13 μM. Performed in 50 mM aqueous HEPES buffer at pH 7.4, T = 25 °C, excitation: 496 nm, emission: 518 nm.

Upon addition of PAMAM G5 into an aqueous solution of calcein buffered at pH 7.4, changes in the spectroscopic signal of the dye clearly indicated that a [calcein•PAMAM] complex was formed (Figure 1). Binding of calcein to the dendrimer caused a red shift in the absorbance spectrum (see Figure S1) and a decrease in absorbance at 500 nm. Fluorescence emission first decreased, then increased to a plateau. This non-monotonic behavior of calcein’s fluorescence emission is due to concentration-mediated self-quenching, which is shared with other xanthene dyes like fluorescein and carboxyfluorescein, as reported previously by our group [24,25].

Fluorescence anisotropy (Figure 1c) measurements were also conducted. Fluorescence anisotropy, i.e., the directional dependence of the polarization of the fluorescence emission when excited with plane-polarized light, is a direct reporter of the binding state of the small dye to the PAMAM macromolecular host. A significant increase in the fluorescence anisotropy signal is indicative of the binding of the small calcein fluorophore to the much larger macromolecular host; the anisotropy reading is high when the small fluorophore is bound to the large host molecule, and low when the dye is free in solution. Since an increase in fluorescence anisotropy could only be ascribed to the formation of a complex between the small fluorophore and the dendrimer, the data shown in Figure 1c conclusively indicates the formation of the postulated [dye•PAMAM] complex sensor.

### 3.2. Binding of Carboxylate to PAMAM G5 Dendrimer (Indicator Displacement Assay)

**Figure 2:**
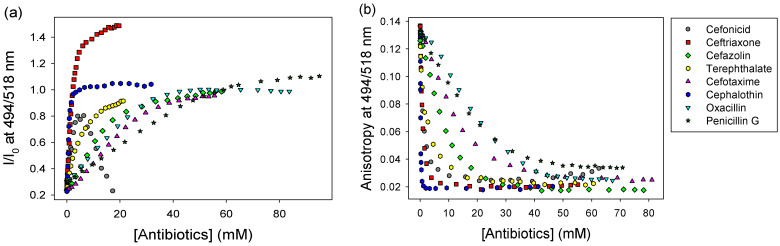
Binding of antibiotics and terephthalate to PAMAM G5 using calcein as an indicator. Titration profiles of analytes of interest into [calcein•PAMAM] complex. (**a**) Fluorescence emission intensity; (**b**) fluorescence anisotropy. [calcein] = 6.36 μM, [PAMAM G5] = 2.13 μM, excitation: 494 nm. Performed in 50 mM aqueous HEPES buffer at pH 7.4, T = 25 °C.

Once we established that we could form the [calcein•PAMAM] complex sensor, we tested the affinity of the antibiotic analytes for PAMAM G5 by introducing them into a solution of the [calcein•PAMAM] sensor. Their binding to PAMAM displaced the calcein dye from the complex and caused its spectroscopic properties to change, indicating the binding event. The resulting fluorescence titration spectra are shown in Figure S2; profiles from these titrations are shown in Figure 2. Fluorescence emission increased as more antibiotic was introduced until, at the end of the titration, the free dye spectrum was recovered, indicating that the dye was in its free form, having been completely displaced from its complex with PAMAM by the bound antibiotics. Similarly, fluorescence anisotropy decreased during titration, showing a clear reversal of the signal associated with the dye binding process; upon addition of antibiotic, the characteristically high anisotropy signal of the bound dye gradually transitioned to the low anisotropy signal of the free dye species, as the bound dye was displaced from the dendrimer by the incoming antibiotic and released back to the bulk solution. Overall, both fluorescence readings, i.e., emission and anisotropy, showed that the antibiotics were binding to the PAMAM dendrimer, and that this binding could be monitored through our indicator displacement approach.

In these preliminary studies, we observed that a high concentration of antibiotics would result in precipitation when added into the sensing complex. The precipitation skewed the absorbance measurements; therefore, these measurements could not be used in these conditions. Fluorescence emission had a higher tolerance for precipitation in this case. Cefonicid (Figure S2e) was an exception; with the addition of cefonicid to the sensing complex, emission intensity increased and then decreased due to the formation of a precipitate. Fortunately, however, the precipitation took place at a higher concentration than that necessary for analyte discrimination, so precipitation did not affect the discrimination results. On the other hand, titration of the monoanionic benzoate was not possible because its affinity for PAMAM and solubility in our working buffer were both too low, so we could not prepare a titrant solution of sufficiently high concentration.

From the profiles shown in Figure 2, almost all analytes showed similar trends during titration; fluorescence emission increased until saturation, and fluorescence anisotropy decreased to a minimum. The analytes’ relative binding affinities to PAMAM could be estimated from these profiles by fitting the profiles to a Langmuir-type saturation binding model (see Supporting Information). The resulting relative affinities, listed in Table 1, can be correlated to the charge state of the analytes; a higher negative charge on the antibiotic resulted in stronger affinity for PAMAM, thanks to stronger electrostatic interactions with the polycationic dendrimer, in accordance with similar trends we previously reported for other anionic guests [18,22]. This trend was particularly clear in the fluorescence anisotropy profiles because anisotropy directly reports on the molar fraction of dye bound to the macromolecular host. In the present set of di- and monoanions, dianionic cephalosporins (cefonicid and ceftriaxone) were found to have the highest affinity for PAMAM dendrimers (*K* ≈ 10^3^), followed by monoanionic cephalosporins (cefazolin and cefotaxime with *K* ≈ 10^2^), then monoanionic penicillins (*K* ≈ 10^1^ − 10^2^). We ascribe the latter difference to the fact that the more hydrophobic cephalosporins establish more favorable hydrophobic interactions with PAMAM, which accounts for their higher observed affinity to the dendrimer compared to the less hydrophobic and more water-soluble penicillins. Interestingly, terephthalate, although also a dianion, displays a lower relative affinity (*K* ≈ 600) than other dianionic antibiotics. Since this smaller anion is more hydrophilic than the dianionic antibiotics, a higher energetic cost is associated with its desolvation, resulting in comparatively lower affinity for the PAMAM host.

**Scheme 4:**
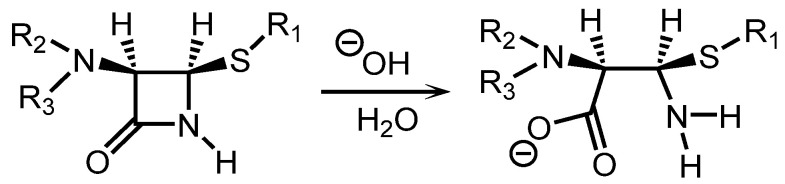
Hydrolysis of β-lactam ring under basic conditions.

Cephalothin was an interesting outlier in the affinity trend mentioned above. In fact, it natively contains only one carboxylate group and should display the lower affinity characteristic of other monoanions; however, it was found to behave more like a dianion. This was due to partial hydrolysis of its β-lactam ring. In fact, cephalothin was found to dissolve in pH 7.4 buffer only sluggishly, so, to speed up the dissolution process, it was first dissolved in basic water (pH 12), and then the resulting solution was brought back to the working pH (pH 7.4). This brief treatment at high pH was sufficient to partially hydrolyze the amide moiety in the strained β-lactam ring and to create a second carboxylate anion (Scheme 4), thereby making cephalothin into a dianion and increasing its observed affinity to a par with other dicarboxylates. This interesting behavior was exploited further, as described below.

The most appropriate concentration of antibiotic to use for discrimination with the [calcein•PAMAM] sensor was determined by visual inspection of the titration profiles shown in Figure 2b; we selected as optimal that concentration of antibiotic at which the anisotropy signal was most different across all target analytes. In this case, the optimal concentration was found to be 5.0 mM. At this concentration, there was no precipitation, so we could measure absorbance as well.

### 3.3. Antibiotics Discrimination at pH 7.4

The antibiotic discrimination study was first carried out in buffered water at pH 7.4 using a 384-well microplate and a plate reader. Each analyte was laid out in 32 replicates and 54 instrumental measurements were collected per sample (see Table S1), resulting in a 54-dimensional dataset. Pattern-based recognition algorithms were then used for data interpretation and dimensionality reduction. Such algorithms included Linear Discriminant Analysis (LDA) [26], which is a clustering technique that can be used to reduce the data complexity and generates a two- or three-dimensional scores plot that can be easily visualized [27,28]. LDA finds the linear combinations of the raw instrumental measurements that minimize the size of the cluster of replicates pertaining to each sample (intracluster distances) while maximizing the distances among clusters belonging to different analytes (intercluster distances), thus providing optimal separation among analyte clusters [29,30]. This method has been used for the discrimination of various analytes, including bacteria [31], proteins [32], wines [33], sugars [34,35], metal ions [36,37], food additives [38], and drugs [39].

The LDA scores plot resulting from this dataset is shown in Figure S4a. Although the clusters are tight and the cephalosporin analytes were well separated, unfortunately, in these conditions, the penicillin analytes were barely separated. Their position very close to the bound dye cluster (see Figure S4b) indicated that, at this concentration, their low affinity for PAMAM dendrimers led to minimal binding of the penicillins to the dendrimer. Most of the dye was therefore still bound to PAMAM, resulting in spectral signatures almost indistinguishable from the bound dye reference standard. Looking at the corresponding loadings plot in Figure S5, differences in absorbance at 350 nm were the main driving force for analyte differentiation. This was due to the significant intrinsic absorption of cephalosporins in this region of the spectrum, rather than to the analytes’ differential interaction with PAMAM. This result was not ideal; relying on the spectral properties of the antibiotics themselves would be far less versatile than the system proposed here, based on their differential interaction with the dendritic polyanion, which could behave as a “universal ligand”. Analytes were not separated well in these conditions, and the system did not make efficient use of the information present in the full dataset. For further improvement, we introduced a sample pre-treatment step to partially hydrolyze the β-lactam ring. Hydrolysis of the amide functionality in these structures would increase the number of carboxylate groups and overall negative charge on the analytes, and therefore increase their affinity for PAMAM dendrimers. This was promising, considering the successful attempt we had made with cephalothin, as discussed above (see Section 3.2 and Figure 2).

### 3.4. Increasing Affinity by Analyte Hydrolysis

We decided to take advantage of our serendipitous observation that hydrolysis of the β-lactam ring, easily achievable in a strongly basic aqueous solution, would expose one more carboxylate moiety in these antibiotics and increase their affinity for our polycationic PAMAM receptor. We expected that each structure would undergo hydrolysis to a different extent, thereby increasing the differences among the antibiotic analytes.

To determine the best conditions for β-lactam ring hydrolysis, we considered pre-treatment at pH 10 and pH 12. Antibiotics were dissolved in a buffer at the pre-treatment pH and the solution was shaken for two minutes before the pH was brought back to 7.4 to stop the hydrolysis and for measurement. Samples pre-treated at pH 10 or pH 12 and then brought back to pH 7.4 were labeled “pH 10–7.4” and “pH 12–7.4”, respectively. Three analytes were studied: monoanionic penicillin G, and dianionic cephalosporins cefazolin and ceftriaxone.

**Figure 3:**
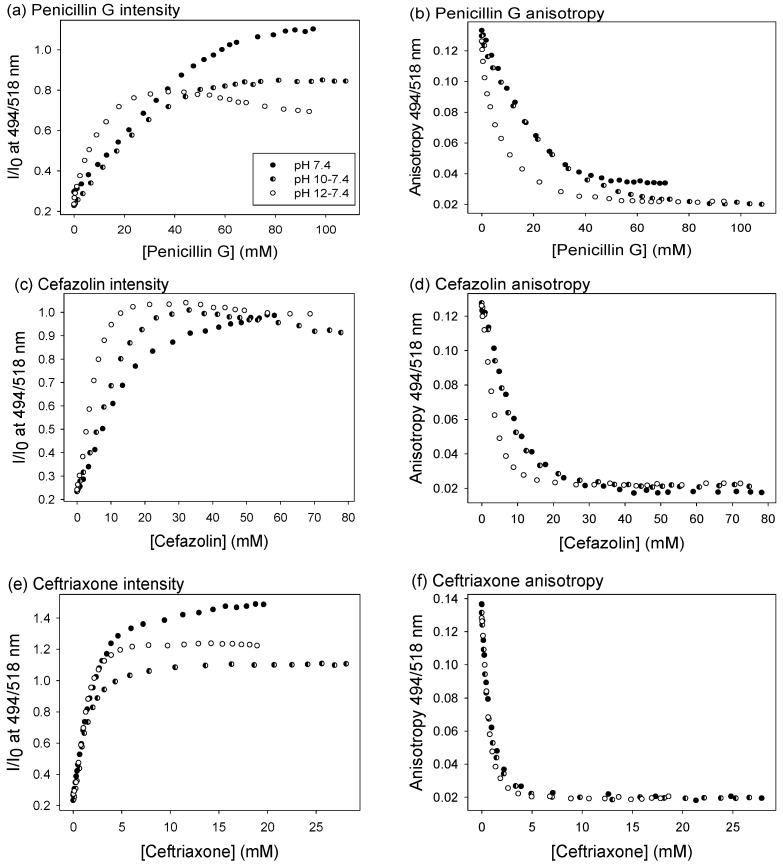
Fluorescence emission profiles from the titration of penicillin G (**a**,**b**), cefazolin (**c**,**d**), and ceftriaxone (**e**,**f**) into [calcein•PAMAM] complex in solutions prepared at different pH (7.4, 10, and 12) and brought back to pH 7.4, corresponding to different levels of β-lactam ring hydrolysis. Left: fluorescence emission; right: fluorescence anisotropy. [calcein] = 6.36 μM, [PAMAM G5] = 2.13 μM, excitation: 494 nm, emission: 518 nm. Performed in 50 mM aqueous HEPES buffer at pH 7.4, T = 25 °C.

Binding titrations conducted with the hydrolyzed samples (in Figure S6) produced results similar to those previously obtained with non-hydrolyzed samples (Figure S2), proving that, even after hydrolysis, analytes could still bind to PAMAM dendrimers as indicated by the displacement of the calcein dye from its complex with the dendrimer. By plotting the fluorescence emission and anisotropy profile obtained from samples hydrolyzed at pH 10 or 12, or not hydrolyzed (Figure 3), we could capture the differences across different working conditions. For penicillin G (monoanion), the sample hydrolyzed at pH 12 clearly had a higher affinity for PAMAM dendrimers, while the sample hydrolyzed at pH 10 and the non-hydrolyzed one were very similar. For cefazolin (monoanion), we noticed that the observed affinity increased with the pre-treatment pH. For ceftriaxone (dianion), all three profiles were very similar. This proved that hydrolysis could increase the affinity of some analytes for PAMAM dendrimers. Furthermore, the variations in behavior among these analytes were advantageous because they would provide one more point of difference among these samples and thus improve the discrimination.

**Figure 4:**
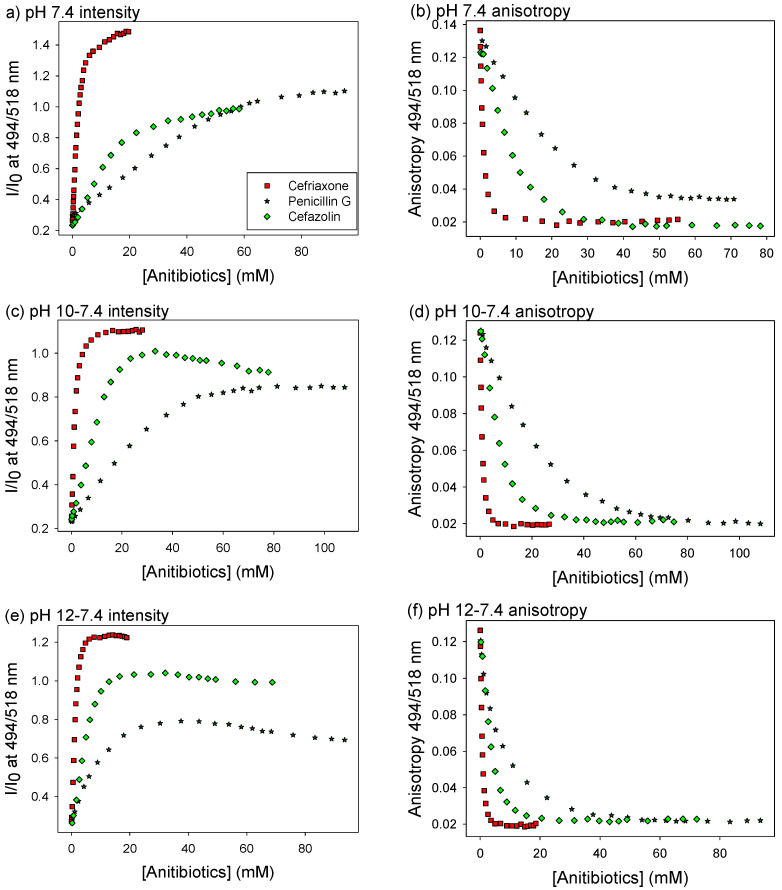
Fluorescence emission profiles from titrations of penicillin G, ceftriaxone, and cefazolin into [calcein•PAMAM] complex in solution. (**a**,**b**) Solutions prepared at pH 7.4; (**c**,**d**) solutions prepared at pH 10 and brought back to 7.4; (**e**,**f**) solutions prepared at pH 12 and brought back to 7.4. Left: fluorescence emission; right: fluorescence anisotropy. [calcein] = 6.36 μM, [PAMAM G5] = 2.13 μM, excitation: 494 nm, emission: 518 nm. Performed in 50 mM aqueous HEPES buffer at pH 7.4, T = 25 °C.

Comparing the behavior of these three analytes when subjected to different pre-treatment pH at 5.0 mM concentration of analyte (Figure 4), the samples prepared at pH 10 and brought back to pH 7.4 gave rise to the largest differences in spectroscopic signal across all target analytes. Therefore, we decided to use “pH 10–7.4” as the working condition to perform further discrimination experiments.

### 3.5. Antibiotic Discrimination on Partially Hydrolyzed Samples

A new discrimination experiment was performed using analytes that had been pre-treated by partial hydrolysis at pH 10 followed by acidification to pH 7.4 for analysis. On a 384-well polystyrene black-wall multiwell plate, we deposited 32 replicates of each analyte (100 μL, 5.0 mM), as well as free and bound dye references, and HEPES buffer as blanks. For each sample, 54 instrumental measurements (see Table S1) were taken by a plate reader, including 30 absorbance wavelengths, 12 fluorescence emission measurements, and 12 fluorescence anisotropy measurements (with different excitation and emission wavelengths combinations). Data acquisition for this extensive training set took roughly 3 h. The resulting spectroscopic measurements collected for each sample generated a multidimensional dataset. To pinpoint the most useful information sources among these measurements for antibiotic discrimination, LDA was used for data processing.

**Figure 5:**
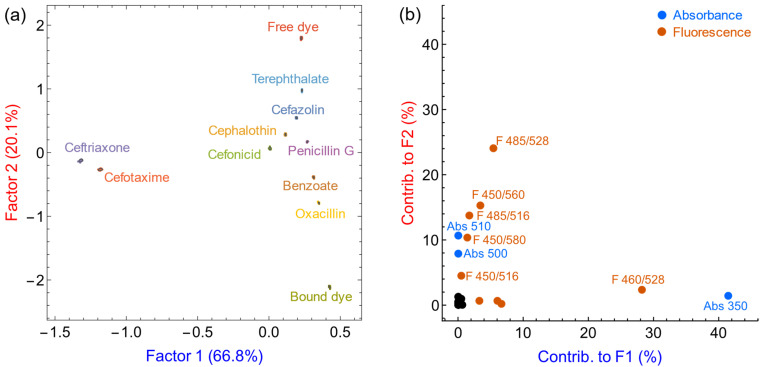
LDA results for qualitative discrimination of nine analytes (two penicillins, five cephalosporins and two reference carboxylates) using [calcein•PAMAM] sensor and hydrolytic sample pre-treatment. (**a**) Scores plot; (**b**) loadings plot. Abs: absorbance, F: fluorescence emission λ_ex_/λ_em_. The black points correspond to instrumental measurements with very low contributions to both LDA factors; these were mostly fluorescence anisotropy measurements that were later removed in further analyses. Analyte solutions were prepared at pH 10 and then brought back to pH 7.4 for β-lactam ring hydrolysis. [calcein] = 6.36 μM, [PAMAM G5] = 2.13 μM, [analytes] = 5.0 mM. Performed in 50 mM aqueous HEPES buffer, T = 25 °C.

The resulting LDA scores plot is shown in Figure 5, with factor 1 and factor 2 representing 66.8% and 20.1% of the overall dataset’s information content, respectively. We were very excited to see that all analyte clusters were well separated with this approach, with larger intercluster distances (clusters were further apart from each other, indicating stronger discriminatory power) and small intracluster distances (clusters were tighter, indicating better reproducibility) compared with the previous results obtained without hydrolytic pre-treatment (see Figure S4). Analytes that had been too close to each other on the scores plot for effective separation and very close to the bound dye reference cluster were now more spread out and further away from the reference cluster. This proved that the hydrolysis process did increase the analytes’ affinity for PAMAM dendrimers, ultimately leading to better differentiation.

The loadings plot shown in Figure 5 shows that, in these conditions, fluorescence measurements contributed more to both factors than without the hydrolysis pre-treatment. Differences in absorbance at 350 nm were still behind the great separation between ceftriaxone and cefotaxime on the one hand, and the rest of the sample clusters along factor 1. However, the quality of the analytical data collected after hydrolytic pre-treatment was considerably improved compared with the non-treated results (compare with Figure S4); in fact, instrumental measurements reporting on properties of the sensing complex (e.g., fluorescence emission measurements) started to contribute more than those related to the intrinsic absorption of the analyte in the UV range (e.g., absorbance at 350 nm), proving the worth of the sensing complex in differentiation of antibiotics analytes. Also, more instrumental measurements contributed to each factor, indicating a more multivariate approach and the capture of multifaceted information. Overall, partial hydrolysis led to much better differentiation results than those obtained previously without pre-treatment (compare with Figure S4). Validation of these results was carried out using bootstrap methods and unbiased PCA analysis [23], which confirmed their consistency and reproducibility (see Figures S7 and S8).

**Figure 6:**
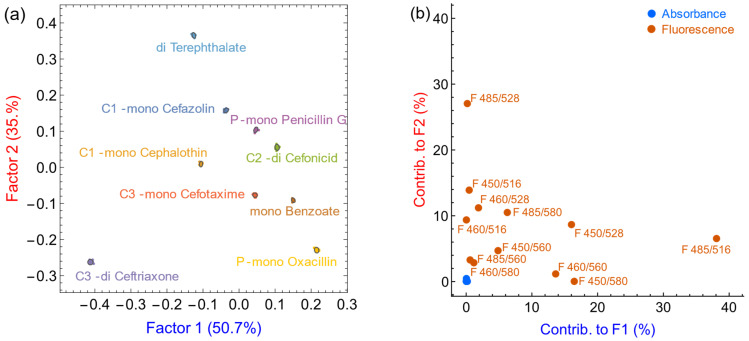
LDA results for qualitative discrimination of nine analytes (two penicillins, five cephalosporins and two reference carboxylates) using [calcein•PAMAM] sensor and hydrolytic sample pre-treatment. (**a**) Scores plot; (**b**) loadings plot. Abs: absorbance, F: fluorescence emission λ_ex_/λ_em_). Analyte solutions were prepared at pH 10 and then brought back to pH 7.4 for β-lactam ring hydrolysis. The same set of raw measurements was used as in Figure 5, but measurements of absorbance in the UV range and dye clusters were removed to improve analyte separation. [calcein] = 6.36 μM, [PAMAM G5] = 2.13 μM, [analytes] = 5.0 mM. Performed in 50 mM aqueous HEPES buffer, T = 25 °C.

In the LDA scores plot, ceftriaxone and cefotaxime clusters can be found on the left side, compressing the rest of the clusters toward the right side of the plot. Since these two cephalosporins had very high intrinsic absorption in the UV range, the huge differences in the UV absorbance reading between these two analytes and the rest overwhelmed other measurements. To better capture smaller differences of the analytes’ interaction with PAMAM dendrimers, we removed the UV absorbance measurements from the dataset. We also decided not to include the reference samples (free dye, bound dye), since we had already extracted as much information as possible from their relative location in our previous analysis (Figure 5a). After re-processing the dataset, the resulting LDA scores plot is shown in Figure 6. The balance of information content between the first two factors was better than before. Even though the intracluster distances were slightly enlarged, the clusters were still tight, and the intercluster distances were larger than previously observed, with a more effective use of space in the scores plot, indicating more effective discrimination. This was a much better result compared to the previous LDA scores plot (Figure 5a). According to the loadings plot in Figure 6, many instrumental measurements did not contribute significantly to the differentiation, including all absorbance measurements and many fluorescence measurements. Therefore, we had an opportunity to further reduce the number of instrumental measurements necessary for the differentiation, leading to an overall simpler process.

**Figure 7:**
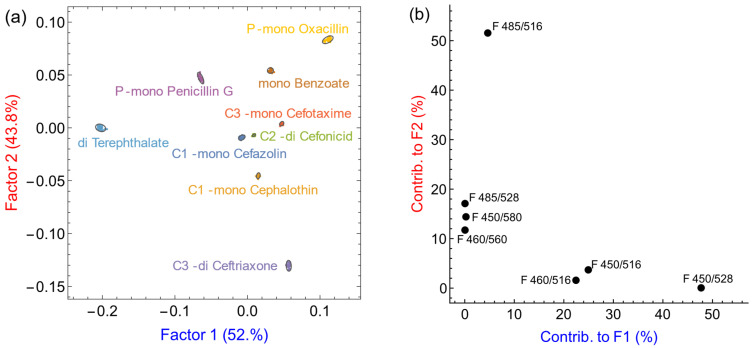
LDA results for qualitative discrimination of nine analytes (two penicillins, five cephalosporins and two reference carboxylates) using [calcein•PAMAM] sensor. (**a**) Scores plot; (**b**) loadings plot. F: fluorescence emission λ_ex_/λ_em_. Analyte solutions were prepared at pH 10 and then brought back to pH 7.4 for β-lactam ring hydrolysis. The same set of raw measurements was used as in Figure 5, but only the seven most important measurements were retained, and dye clusters were removed clarity. [calcein] = 6.36 μM, [PAMAM G5] = 2.13 μM, [analytes] = 5.0 mM. Performed in 50 mM aqueous HEPES buffer, T = 25 °C.

Based on the previous LDA loadings plot (Figure 6), we tried different combinations of fluorescence measurements; in each successive attempt we removed the least contributing measurement according to the loadings plot, until further removal impaired the differentiation. Ultimately, the most relevant and information-rich instrumental measurements were reduced to seven, all of which were fluorescence emission measurements with different excitation and emission wavelength combinations (shown in Figure 7). The resulting LDA scores plot is shown in Figure 7 (left); this showed balance between the factor contributions, and good discrimination among analytes. By retaining seven fluorescence emission measurements, the acquisition time for the construction of such training sets as the ones shown here could be halved, from the original 2 h to around 1 h. Although the process of construction of the training sets described here would not be repeated each time an unknown sample is measured, nevertheless, such calibrations would still have to be repeated periodically in practical use (e.g., daily, weekly), so it was still important to minimize the repetitive effort required. Besides, acquiring low-information-content measurements would increase the noise in the system while contributing little to the useful discriminatory power.

### 3.6. Limit of Discrimination

**Figure 8:**
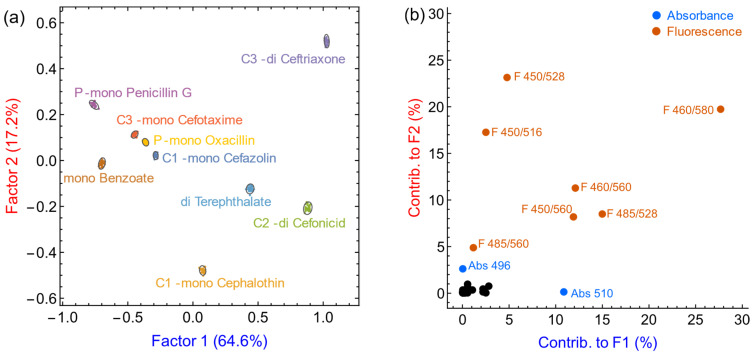
LDA results to estimate the limit of discrimination of nine analytes (two penicillins, five cephalosporins and two reference carboxylates) using [calcein•PAMAM] sensor. (**a**) Scores plot; (**b**) loadings plot. Abs: absorbance, F: fluorescence emission λ_ex_/λ_em_. The black points correspond to instrumental measurements with very low contributions to both LDA factors; these were mostly fluorescence anisotropy measurements that were later removed in further analyses. Analyte solutions were prepared at pH 10 and then brought back to pH 7.4 for β-lactam ring hydrolysis. Measurements of absorbance at UV range and dye clusters were removed for better separation of analyte clusters. [calcein] = 6.36 μM, [PAMAM G5] = 2.13 μM, [analytes] = 1.0 mM. Performed in 50 mM aqueous HEPES buffer, T = 25 °C.

Finally, an experiment was performed to determine the limit of discrimination by reducing the analyte concentration to 1.0 mM, while the rest of experimental conditions and data processing procedures remained the same as the results shown above in Figure 6. The LDA scores plot shown in Figure 8 resulted from a dataset in which UV absorbance measurements were removed. Once again, reference clusters were also removed for clarity. Although the analyte clusters were all separated, the intercluster distance became very small, indicating poor discriminatory power, and the distribution of information between factors 1 and 2 became unbalanced. It was likely that lowering the analyte concentration further would prevent successful discrimination. Therefore, the limit of discrimination of this sensing system for antibiotics was estimated to be not lower than 1.0 mM as a conservative estimate.

## 4. Conclusions

We developed a hydrolytic pre-treatment method for β-lactam antibiotics in the penicillin and cephalosporin family that contain carboxylate groups. This method results in partial hydrolysis of the lactam ring, revealing an additional anionic carboxylate group and increasing their binding affinity towards PAMAM dendrimers, which are a family of commercially available, macromolecular water-soluble polycations that have been used extensively in our group to bind to anionic substrates in water through non-covalent interactions. We monitored these interactions through an indicator displacement assay based on calcein, which is an efficient, commercially available organic chromophore and fluorophore. Taking advantage of the discriminatory power of the [calcein•PAMAM] supramolecular sensor, combined with a pattern-based sensing approach, two penicillin analytes, five cephalosporin analytes, and two reference analytes of interest were successfully differentiated through absorbance, fluorescence emission, and anisotropy measurements after β-lactam ring hydrolysis. A common and lightweight data clustering method, linear discriminant analysis, was used for data processing. The limit of discrimination of the system for these antibiotics was estimated to be not lower than 1.0 mM.

## Figures and Tables

**Table 1:** Relative binding affinities of antibiotics to PAMAM G5, calculated by nonlinear fitting of a Langmuir-type saturation binding model to the anisotropy profiles from Figure 2. See Figure S3 in the Supporting Information for detailed fitting results.

Antibiotic	Family	Nominal Charge	Relative Affinity *K*
Cefazolin	cephalosporin	monoanion	115 ± 7
Cefotaxime	cephalosporin	monoanion	86 ± 8
Cephalothin	cephalosporin	monoanion	5068 ± 262 ^1^
Penicillin G	penicillin	monoanion	81 ± 9
Oxacillin	penicillin	monoanion	58 ± 5
Cefonicid	cephalosporin	dianion	1073 ± 118
Ceftriaxone	cephalosporin	dianion	1251 ± 97
Terephthalate	reference	dianion	590 ± 102

^1^ Sample was insoluble at pH 7.4, so it was partially hydrolyzed in basic conditions to improve its solubility before measurement; see discussion below.

## Data Availability

Data generated in this study are available from the corresponding author.

## Supplementary Materials

The following are available online at https://www.mdpi.com/article/10.3390/s21196384/s1. Figure S1: Binding between PAMAM G5 and calcein by titrating PAMAM G5 into calcein, Figure S2: Fluorescence emission spectra from titrating analytes into [calcein•PAMAM] sensor in solution, Figure S3: fitting of anisotropy titration profiles to obtain relative affinity constants, Figure S4: LDA scores plot for qualitative discrimination of nine analytes using [calcein•PAMAM] sensor, Figure S5: LDA loadings plot for qualitative discrimination of nine analytes using [calcein•PAMAM] sensor, Figure S6: Fluorescence emission spectra of titrating penicillin G, cefazolin, and ceftriaxone into [calcein•PAMAM] complex in solution, Table S1: Loadings for instrumental measurements used for the qualitative discrimination of antibiotics using [calcein•PAMAM] sensor, Figure S7 and Figure S8: bootstrap validation of chemometrics results.
