# Supplementary material for: Beta-Lactam Antibiotic Discrimination Using a Macromolecular Sensor in Water at Neutral pH
Source: Sensors (Basel). 2021 Sep 24;21(19):6384. doi: 10.3390/s21196384 (PMC8512602; doi:10.3390/s21196384)
Supplement: Supplementary file 1 [file sensors-21-06384-s001.zip › sensors-1338857-supplementary.pdf]

Beta-Lactam Antibiotic Discrimination Using a Macromolecular Sensor in Water at Neutral pH

Yifei Xu [1] and Marco Bonizzoni [1,2,*]

[1] Department of Chemistry and Biochemistry, The University of Alabama, Tuscaloosa, AL 35487, USA; xxu56@crimson.ua.edu
[2] Alabama Water Institute, The University of Alabama, Tuscaloosa, AL 35487, USA
* Correspondence: marco.bonizzoni@ua.edu

Contents:

Absorption and fluorescence spectra from the binding between PAMAM G5 and calcein..S2

Fluorescence emission spectra for antibiotics titrated into [calcein•PAMAM] sensor at pH 7.4 ....S2

Determination of relative affinities of antibiotics for PAMAM..S5

Antibiotic discrimination at pH 7.4 ....S6

LDA loadings for discrimination using [calcein•PAMAM] S8

Fluorescence spectra for antibiotics binding after pre-hydrolysis S9

Bootstrap validation of the chemometrics results S9

References S11

Absorption and fluorescence spectra from the binding between PAMAM G5 and calcein

We have previously shown that calcein can bind with PAMAM G5 in buffered water at pH 7.4 [1]. Here in Figure S1 shows the absorbance and fluorescence spectra of titrating PAMAM G5 into calcein solution. 2 mL of 6.36 μM of calcein in 50 mM aqueous HEPES buffer at pH 7.4 was put into a 1 cm quartz cuvette, then aliquots of a PAMAM G5 solution also containing 6.36 μM of calcein were titrated into the cuvette. During the titration process, the calcein concentration remained the same while the PAMAM concentration increased. The addition of calcein in both titrant and cuvette solutions was done to avoid optical changing due to dilution. The corresponding profiles are shown in Figure 1 in the manuscript. Upon the addition of PAMAM G5, the absorbance peak of calcein shifted towards the red, and fluorescence emission first decreased sharply, then increased to a plateau.

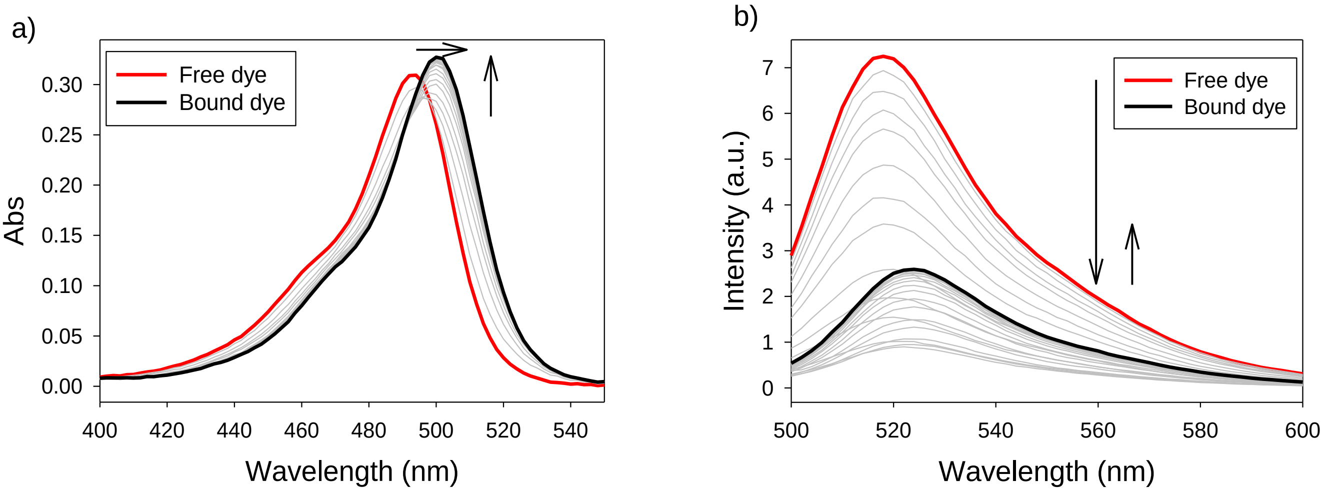

Figure S1. Binding between PAMAM G5 and calcein monitored by titrating PAMAM G5 into calcein. a) Absorbance spectra; b) Fluorescence emission spectra. [calcein] = 6.36 μM, [PAMAM G5] = 2.13 μM, excitation: 494 nm. Performed in 50 mM aqueous HEPES buffer at pH 7.4, T = 25 °C.

Fluorescence emission spectra for antibiotics titrated into [calcein•PAMAM] sensor at pH 7.4

We tested the affinity of the antibiotic analytes for PAMAM G5 by introducing them into the [calcein•PAMAM] sensor, to see if they could displace the calcein dye from its complex. The fluorescence emission titration spectra are shown in Figure S2 while titration profiles are shown in Figure 2 in the manuscript. Fluorescence emission increased as more antibiotics were introduced, and at the end of titration, the emission spectra looked very similar to the free dye spectra. Since both PAMAM and analytes were not fluorescent, this indicated that the dye was in its free form at the end of the titration. Note that in Figure S2e, the introduction of cefonicid first caused a florescence increase, followed by a decrease upon further additions. High concentrations of cefonicid caused a precipitate to form. Fortunately, in our further studies cefonicid concentration was never as high, so precipitation was not a problem. Overall, fluorescence emission measurements showed that the antibiotics were able to displace the calcein dye from its complex, and that this process was accompanied by a measurable change in the spectroscopic signal of the solution.

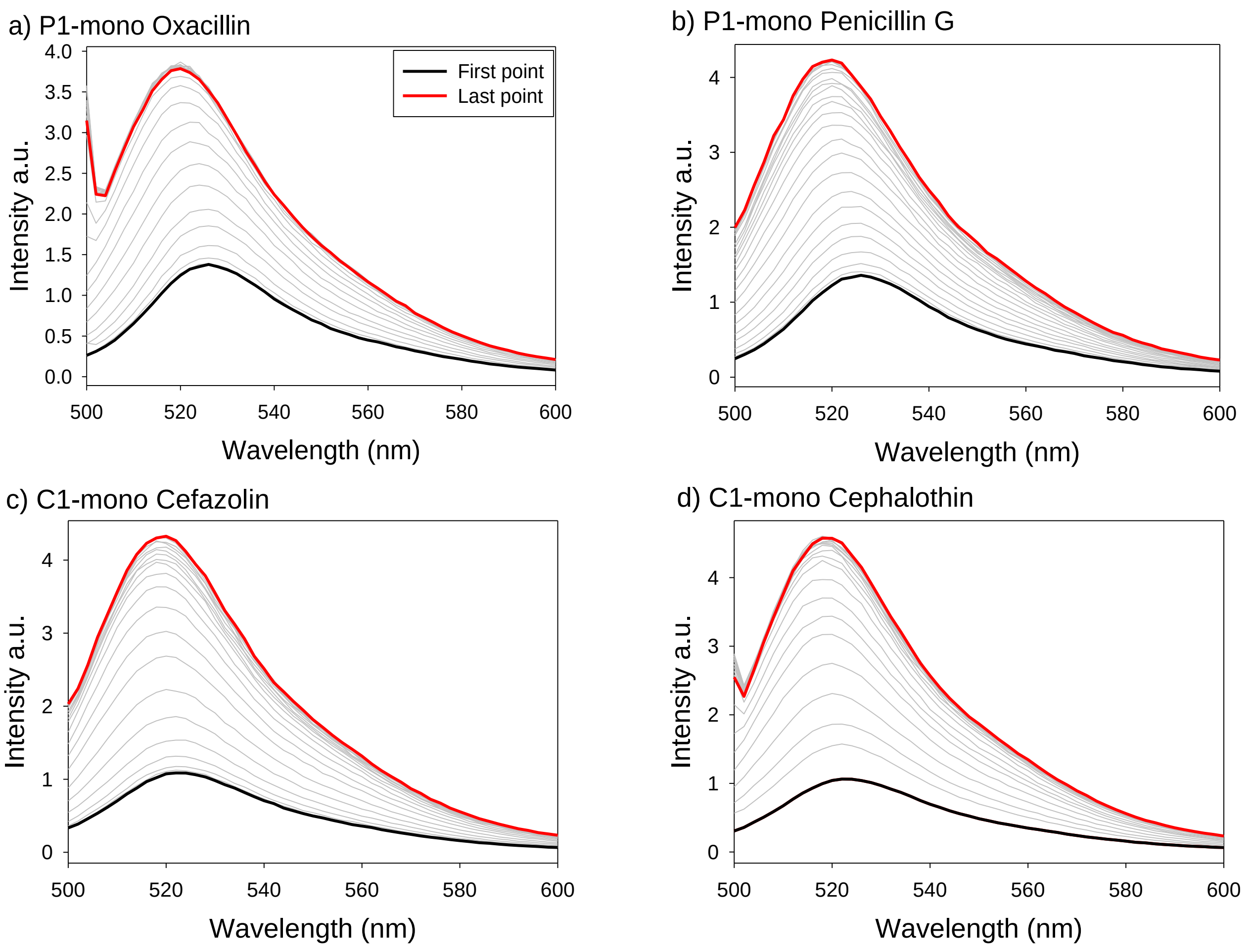

Continued on the next page.

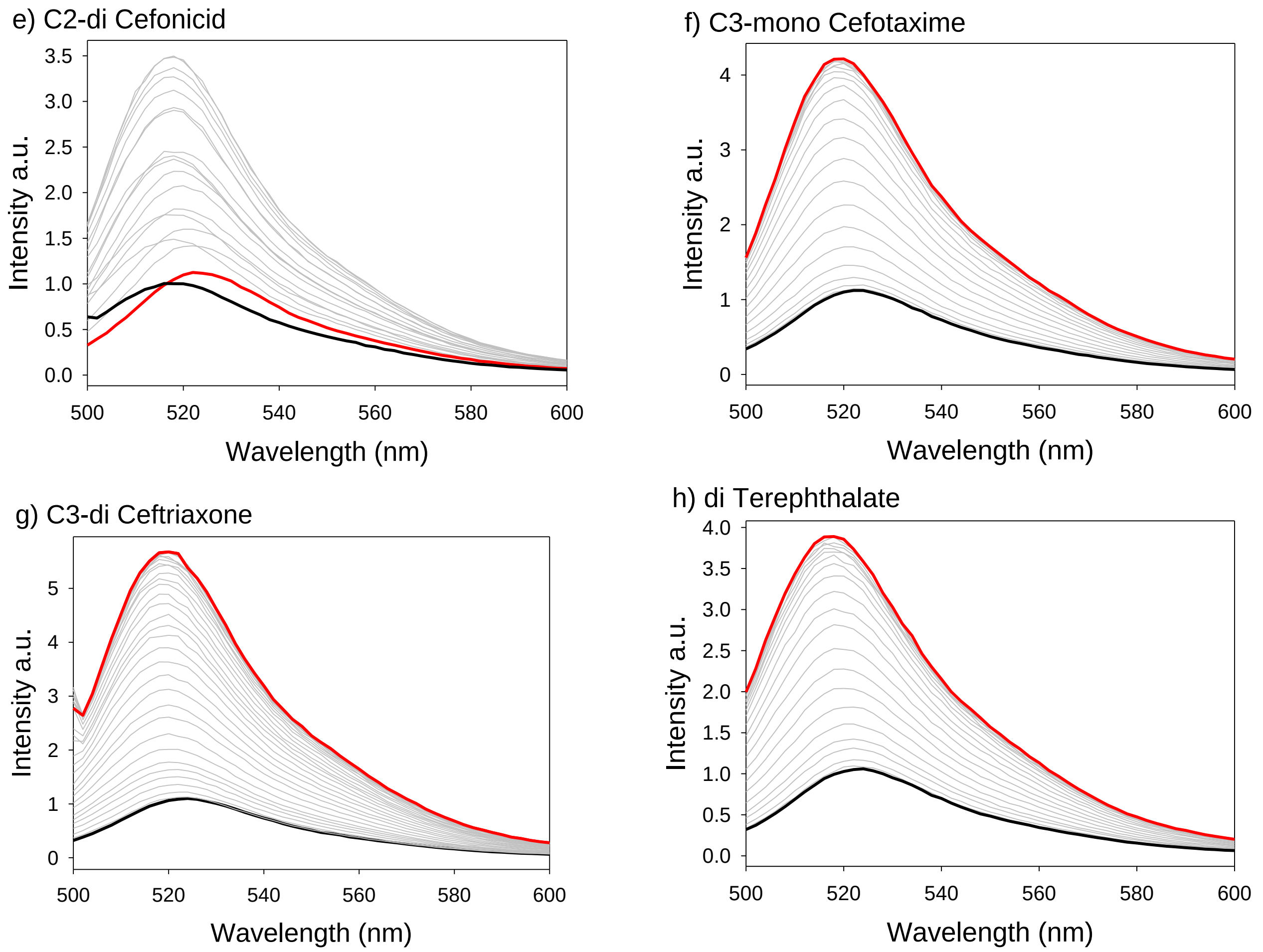

Figure S2. Fluorescence emission spectra obtained upon titrating analytes into a solution containing the [calcein•PAMAM] sensor. a) Oxacillin, b) Penicillin G, c) Cefazolin, d) Cephalothin, e) Cefonicid, f) Cefotaxime, g) Ceftriaxone, h) Terephthalate. The spectrum in black represents the first titration point; the one in red is the last titration point; the spectra in gray represent the intermediate titration points. [calcein] = 6.36 μM, [PAMAM G5] = 2.13 μM, excitation: 494 nm. Performed in 50 mM aqueous HEPES buffer at pH 7.4, T = 25 °C.

Determination of relative affinities of antibiotics for PAMAM

A generic Langmuir-type saturation binding model (shown below) was fitted to the fluorescence anisotropy binding profiles for each antibiotic obtained upon binding to PAMAM G5 (i.e., the data shown in Figure 2b in the manuscript) using a nonlinear least-squares approach. Even with no assumptions on the stoichiometry of interaction and ignoring the dye dissociation equilibrium, this approach yields apparent binding constants that can be used to estimate the relative strength of the antibiotics' interactions with the PAMAM dendrimer.

$$\theta = \frac{\rho_{max}}{1 + K[\mathrm{A}] + K^2[\mathrm{A}]^2} + \rho_{min}$$

In the model above, θ = degree of saturation ($0 \le \theta \le 1$); K = apparent affinity constant; [A] = stoichiometric concentration of added antibiotic; ρ_{max} = initial experimental value of the fluorescence anisotropy signal, in the absence of antibiotic; ρ_{min} = lowest experimental value attained by the anisotropy signal through the titration.

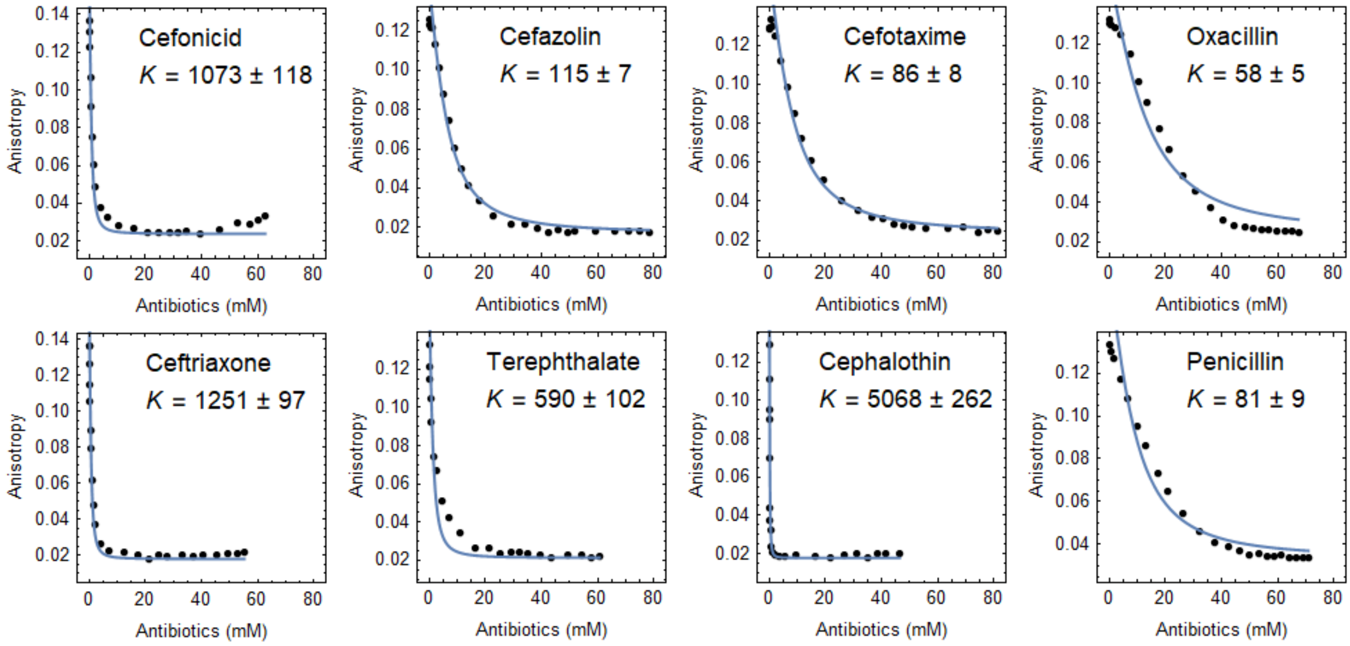

Figure S3. Results from nonlinear least-squares fitting of the fluorescence anisotropy binding profiles of antibiotics to PAMAM G5 in 50 mM aqueous buffer at pH 7.4, T = 25C. Black points= experimental data; blue lines = fitted curves.

Antibiotic discrimination at pH 7.4

On a 384-well polystyrene black-wall multiwell plate with clear bottom, we deposited 32 replicates of each analyte (100 µL, 5.0 mM, prepared in buffer at pH 7.4), as well as free dye and bound dye references, and 50 mM HEPES buffer as blanks. For each sample, 54 instrumental measurements (see Table S1) were taken by a BioTek Synergy II multimode plate reader, including 30 absorbance wavelengths, 12 fluorescence emission measurements and 12 fluorescence anisotropy measurements (with different excitation and emission wavelengths combinations) in a 3-hour period. Multiple optical measurements collected for each sample generated a multidimensional dataset. Linear discriminant analysis (LDA) was used for further data processing.

The LDA scores plot of this dataset is shown in Figure S4: a scores plot of the full analyte set is shown on the left, and on the right is an enlargement of the gray rectangular area of the plot on the left. Factor 1 contained 68.8% of the original information, while factor 2 contained 16.6% of the original information; the balance between the two factors was not ideal but still acceptable. Considering that each sample was measured in 32 replicates, the clusters were very tight, and all the cephalosporin clusters were well separated, indicating that this system worked well for cephalosporin discrimination. However, even though the two penicillin clusters were separated, their intercluster distance was very small. A similar situation was found for benzoate and terephthalate. Considering that the clusters were all very close to the bound dye reference, this could be caused by their low affinity for PAMAM dendrimer. Therefore, at the concentration of 5.0 mM, these analytes could only displace a small amount of dye from its complex with PAMAM. With a majority of the calcein dye being bound to PAMAM dendrimer, these analyte solutions all "looked like" the bound dye spectroscopically and could not be discriminated from each other.

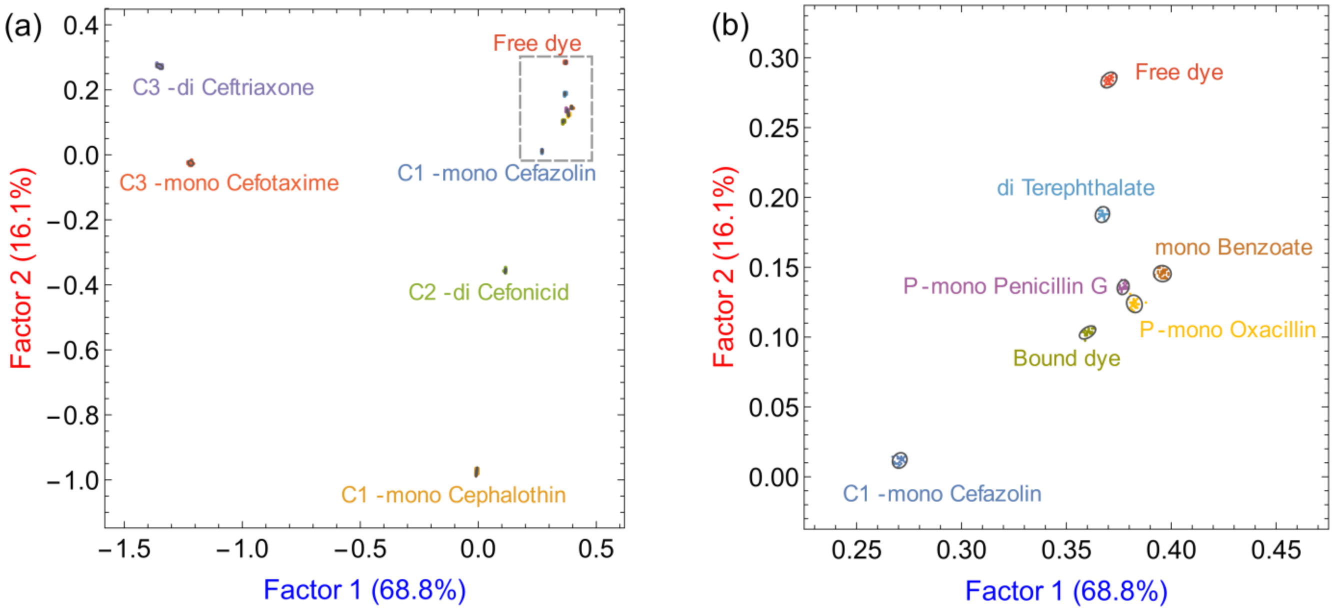

Figure S4. LDA scores plot for qualitative discrimination of nine analytes (two penicillins, five cephalosporins and 2 reference carboxylates) using [calcein•PAMAM] sensor. a) Full scale of the scores plot; b) An enlargement of the rectangular area highlighted in (a). [calcein] = 6.36 µM, [PAMAM G5] = 2.13 µM, [analytes] = 5.0 mM. Performed in 50 mM aqueous HEPES buffer at pH 7.4, T = 25 °C.

The loadings plot corresponding to the scores plot from Figure S4 is shown in Figure S5. The absorbance at 350 nm was the main contributor to both factor 1 and factor 2. This was the reason that ceftriaxone and cefotaxime were very clearly separated from the rest of the clusters along factor 1: in fact, they have very high intrinsic absorption in the UV range, especially around 350 nm. Similarly, cephalothin was far away from the rest of the analytes along factor 2 because it also has intrinsic absorption in the UV range, only not as high as the other two. Although fluorescence measurements did contribute to both factors, at least to some extent, they were still insignificant compared with absorbance at 350 nm. The current situation did not capture much of the differential information in the system, instead primarily responding to a single-variate stimulus, i.e., the absorbance in the UV region.

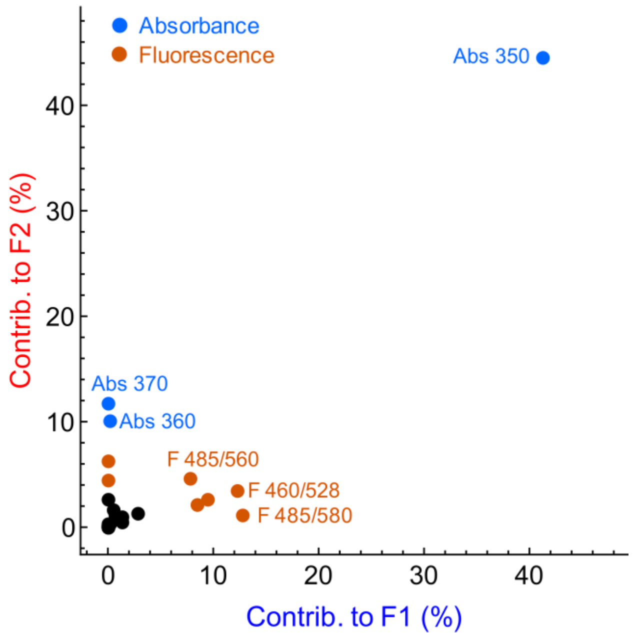

Figure S5. LDA loadings plot for qualitative discrimination of nine analytes (two penicillins, five cephalosporins and two reference carboxylates) using [calcein•PAMAM] sensor (same dataset as the scores plot shown in Figure S4). Abs: absorbance, F: fluorescence emission $\lambda_{ex}/\lambda_{em}$. [calcein] = 6.36 µM, [PAMAM G5] = 2.13 µM, [analytes] = 5.0 mM. Performed in 50 mM aqueous HEPES buffer at pH 7.4, T = 25 °C.

Table S1. Loadings for instrumental measurements used for the qualitative discrimination of antibiotics using [calcein•PAMAM] sensor. Fn% (columns) represents the percent of the information explained by Factor n that is provided by each raw measurement (rows). The numbers correspond to Figure 3 in the manuscript.

**Variable type**	**Wavelength (nm)**	**F1%**	**F2%**	**F3%**
Absorbance	350	41	44	8
Absorbance	360	0	10	2
Absorbance	370	0	12	2
Absorbance	380	0	3	1
Absorbance	390	0	0	0
Absorbance	400	0	0	0
Absorbance	410	0	0	0
Absorbance	420	0	0	0
Absorbance	430	0	0	0
Absorbance	440	0	0	0
Absorbance	450	0	0	0
Absorbance	460	0	0	0
Absorbance	470	0	0	0
Absorbance	480	0	0	0
Absorbance	486	0	0	0
Absorbance	492	0	0	0
Absorbance	494	0	0	3
Absorbance	496	0	0	0
Absorbance	500	0	0	1
Absorbance	510	0	0	7
Absorbance	520	0	0	0
Fluorescence emission	450/516	1	1	0
Fluorescence emission	450/528	9	3	4
Fluorescence emission	450/560	8	2	0
Fluorescence emission	450/580	0	2	0
Fluorescence emission	460/516	0	6	46
Fluorescence emission	460/528	12	3	2
Fluorescence emission	460/560	1	1	1
Fluorescence emission	460/580	0	4	1
Fluorescence emission	485/516	1	0	2
Fluorescence emission	485/528	3	1	10
Fluorescence emission	485/560	8	5	7
Fluorescence emission	485/580	13	1	0
Fluorescence anisotropy	450/528	0	0	0
Fluorescence anisotropy	450/560	0	0	0
Fluorescence anisotropy	450/580	0	0	0
Fluorescence anisotropy	460/528	0	0	0
Fluorescence anisotropy	460/560	0	0	0
Fluorescence anisotropy	460/580	0	0	0
Fluorescence anisotropy	485/528	0	0	0
Fluorescence anisotropy	485/560	0	0	0
Fluorescence anisotropy	485/580	0	0	0

For fluorescence emission, the wavelength pairs refer to excitation and emission filters, respectively. Wavelength selection was achieved through bandpass filters with the following characteristics: 450 ± 25 nm; 460 ± 20 nm; 485 ± 10 nm; 516 ± 10 nm; 528 ± 10 nm; 560 ± 20 nm; 580 ± 25 nm.

Fluorescence spectra for antibiotic binding after hydrolytic pre-treatment

The optimal pre-treatment pH for pre-hydrolysis was determined using penicillin G, cefazolin, and ceftriaxone. These were pre-treated at pH 10 ("pH 10-7.4") or pH 12 ("pH 12-7.4") then brought to pH 7.4. The corresponding binding profiles are shown in Figure 5 and Figure 6 in the manuscript.

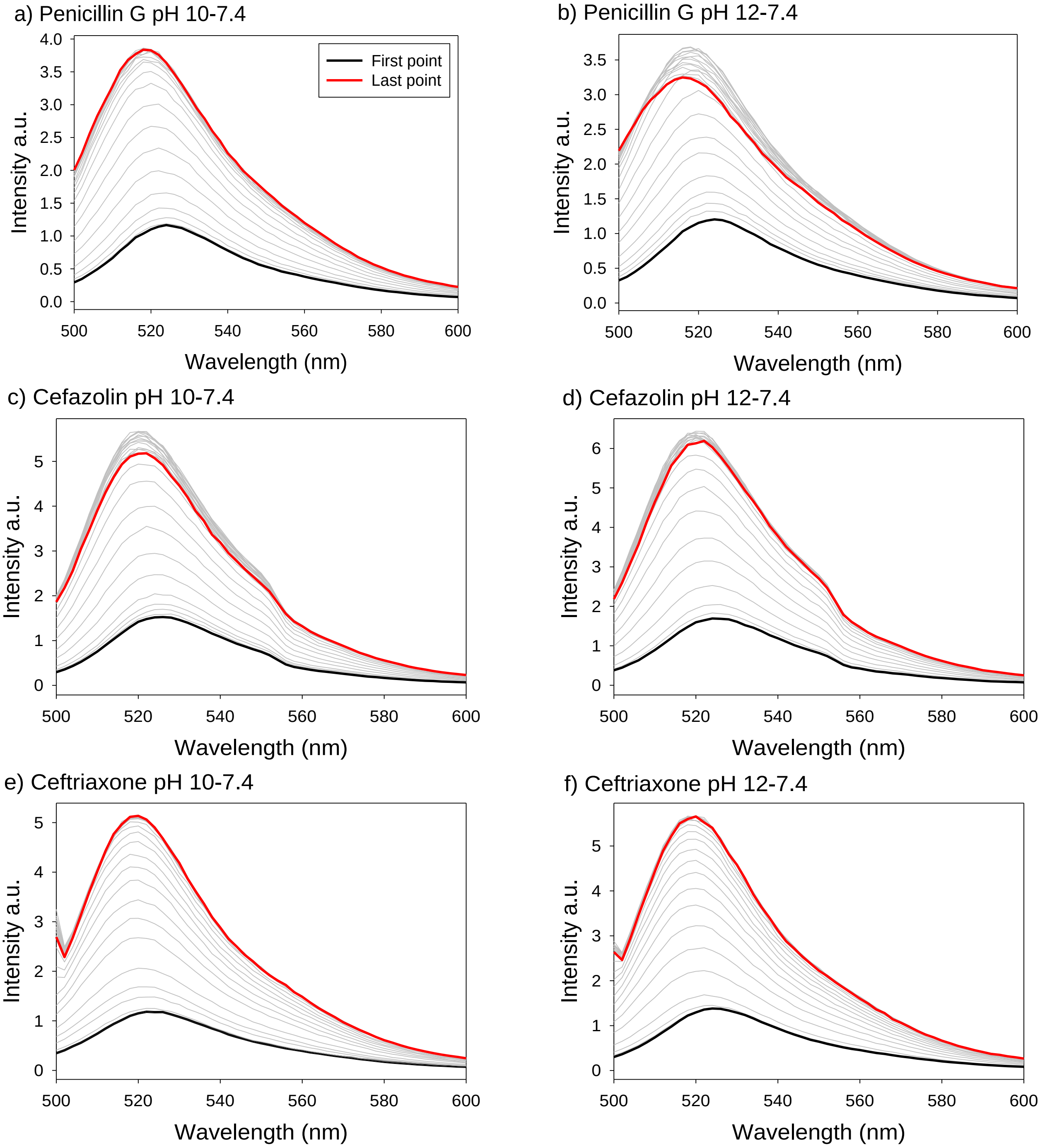

Figure S6. Fluorescence emission spectra from titrations of pre-hydrolyzed analytes into [calcein•PAMAM]. Analytes were partially hydrolyzed for two minutes at pH 10 (left) or pH 12 (right), then brought to pH 7.4 for measurement. [calcein] = 6.36 μM, [PAMAM G5] = 2.13 μM, excitation: 494 nm. Performed in 50 mM aqueous HEPES buffer at pH 7.4, T = 25 °C.

Bootstrap validation of the chemometrics results

After outlier removal, we implemented bootstrap validation [2], by splitting the complete dataset (all measurements, same dataset as Figure S4) into two subsets of similar dimensions, each one containing a representative subsample of the whole, with roughly

half as many replicates as the complete set. The first subset (the *training* set) was subjected to principal component analysis (PCA) but, in addition to the PCA scores, the eigenvectors from the analysis were also retained. The second subset (the *validation* set) was first standardized, then projected onto the component space of the training set, using the previously obtained eigenvectors, to obtain its own PCA scores. The two subsets were then plotted together. Excellent overlap between corresponding analyte clusters from the training and validation sets was observed (Figure S7), which validated the repeatability and consistency of the pattern-based analysis results.

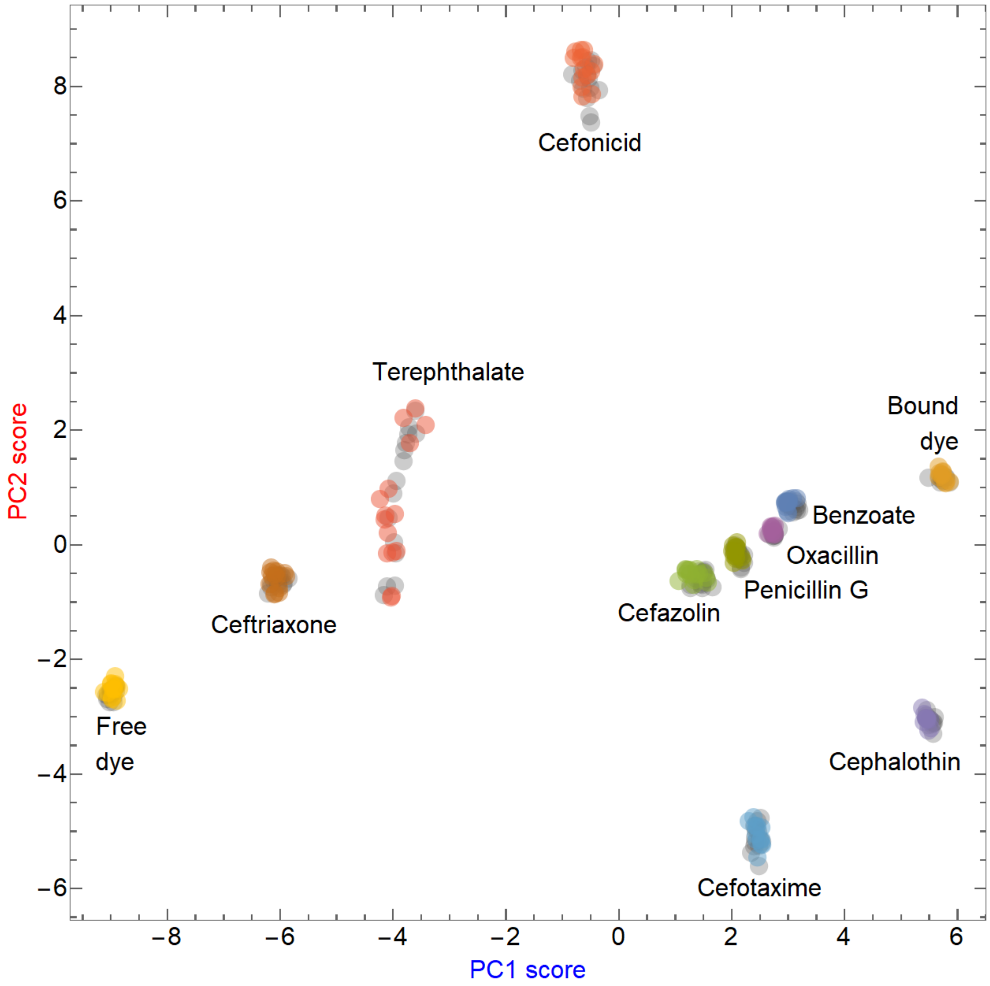

Figure S7. A representative results from PCA-based bootstrap validation of the complete dataset. The points in gray belong to the training set; the colored points belong to the validation set. The excellent overlap between corresponding clusters in the training and validation sets indicates successful validation of the patter-based approach.

The same process was repeated hundreds of times, each time algorithmically selecting a different random choice of training and validation samples, with similar results. Twelve representative results from such split-and-test cycles are shown below in Figure S8.

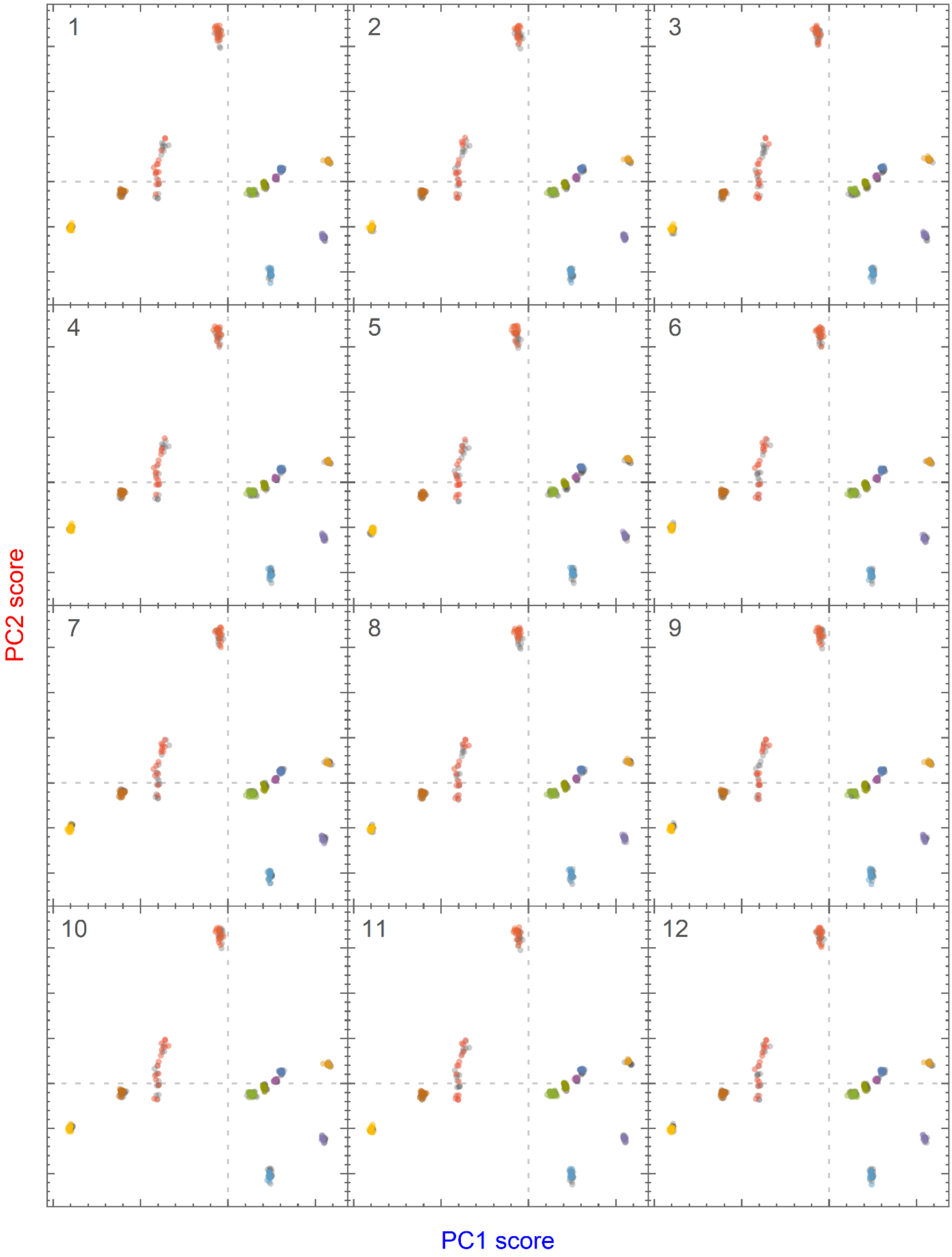

Figure S8. Multiple results from PCA-based bootstrap validation of the complete dataset demonstrating that the excellent overlap between corresponding sample clusters in the training and validation sets did not depend on the choice of samples in these sets.

Reference

1. Xu, Y.; Bonizzoni, M. Discrimination and quantitation of biologically relevant carboxylate anions using a [dye • PAMAM] complex. *Sensors* **2021**, *21*, 3637, doi:10.3390/s21113637.

2. Westad, F.; Marini, F. Validation of chemometric models—A tutorial. *Anal. Chim. Acta* **2015**, *893*, 14–24, doi:10.1016/j.aca.2015.06.056.
